# Left Ventricle Hypertrophy, Dilatation and Ejection Fraction Changes Before and After Kidney Transplantation

**DOI:** 10.4021/cr246w

**Published:** 2013-03-08

**Authors:** Hamid Tayebi-Khosroshahi, Mohsen Abbasnezhad, Afshin Habibzadeh, Masumeh Bakhshandeh, Parastoo Chaichi

**Affiliations:** a Department of Nephrology, Tabriz University of Medical Sciences, Tabriz, Iran; b Department of Cardiology, Cardiovascular Research Center, Tabriz University of Medical Sciences, Tabriz, Iran; c Medical Philosophy and History Research Center, Tabriz University of Medical Sciences, Tabriz, Iran

**Keywords:** End stage renal disease, Renal transplantation, Echocardiography

## Abstract

**Background:**

Patients with end stage renal disease (ESRD) are at risk of complications in different organs including cardiovascular system. Renal transplantation is the best choice in these patients which diminishes these complications. It is observed that after renal transplantation, cardiac parameters have appropriate improvement. Current study evaluates echocardiographic findings in renal transplant recipients before and after kidney transplantation.

**Methods:**

In an analytic cross sectional study, 30 patients (50% male, mean age of 45.57 ± 13.32 years) with ESRD who underwent renal transplantation were studied. All patients had echocardiographic studies after the last dialysis before and 6 months after transplantation. Echocardiographic study was done by Color Doppler two dimension methods and left ventricle ejection fraction was measured by Simpson method. All echocardiograms before and after transplantation were interpreted by the same cardiologist.

**Results:**

Mean left ventricle ejection fraction before and after renal transplantation was 53.83±10.14% and 57.33±4.49%, respectively (P = 0.09). Left ventricle hypertrophy, mitral regurgitation and tricuspid regurgitation existed in 46.7%, 76.7% and 33.3% respectively, which was improved in 30%, 50% and 33.3% after renal transplantation.

**Conclusion:**

According to the results of current study it is suggested that renal transplantation could improve left ventricle parameters in patients with end stage renal disease.

## Introduction

End-stage renal disease (ESRD) is considered as one of the most important diseases with a great burden on health care systems. Complications of ESRD on various organs, especially cardiovascular system, are noticeable [1]. Left ventricular hypertrophy (LVH) is a well-established marker of cardiovascular risk in the general population [2]. LVH, which occurs in response to volume and pressure overload, is the prevalent cardiovascular finding in the patients with ESRD, including renal transplant patients (50-70%) [3, 4].

Renal transplant is the most acceptable treatment modality for the patients with ESRD, which improves some complications of renal failure such as chronic uremia and volume overload [5, 6]. It is reported that successful renal transplantation has positive effects on ventricular hypertrophy and could regress LVH and improve LVEF during the first year after transplantation [6-10].

There is little information about short term echocardiographic changes after renal transplantation. In this study we aim to evaluate echocardiographic findings in renal transplant recipients before and 6-months after renal transplantation.

## Methods

In this retrospective study, 30 patients with ESRD undergone renal transplantation during 2009 - 2011 in Imam Reza Hospital, Tabriz, Iran were evaluated. Exclusion criteria were patients with acute renal rejection, chronic allograft nephropathy with progressively decreased renal function in the first three post-transplant months, ones with heart failure and older than 70 years or younger than 16 years. All patients had received living related kidney grafts. All patients with available echocardiograms before and 6 month after transplantation were included. All evaluated patients were on haemodialysis treatment before renal transplantation. The study protocol was approved by the institutional ethics review board.

Echocardiographic analysis was performed by use of M mod and two-dimensional Doppler apparatus. Due to lack of proper reports of echocardiography before the transplantation, all findings were recorded as qualitative measures and only left ventricle ejection fraction (LVEF) was quantitatively measured.

### Data analysis

Statistical analyses were performed using the Statistical Package for Social Sciences, version 16.0 (SPSS, Chicago, Illinois). Continuous values were expressed as mean ± standard deviation and categorical variables were expressed as percentages. The categorical parameters were compared by χ^2^ tests or Fisher’s exact test. The continuous variables before and after transplantation were compared by paired samples t-tests. A P value < 0.05 was considered significant.

## Results

In this study 30 CRF patients undergone renal transplantation were studied, 15 cases (50%) were male and 15 cases (50%) were female with mean age of 45.36 ± 13.32 years (range 17 - 69 years).

In this study we evaluated most cases qualitatively. Echocardiographic findings before and after renal transplantation are shown in Table 1. The prevalence of all findings was reduced after transplantation, but only mitral regurgitation had significant reduction. LVEF was non-significantly increased after transplantation.

**Table 1:** Echocardiographic Findings Before and After Renal Transplantation

	Before transplantation	After transplantation	P value
LVEF	53.83±10.14%	57.33±4.49%	0.09
LVH	14 (46.7%)	8 (26.7%)	0.18
LV dilation	1 (3.3%)	1 (3.3%)	-
MR	23 (76.7%)	10 (33.3%)	0.002*
mild	13	10	-
moderate	9	0	
severe	1	0	
TR	10 (33.3%)	3 (10%)	0.057
mild	6	3	-
moderate	3	0	
severe	1	0	

* P is two-tailed significant. LVEF: Left ventricle ejection fraction; LVH: Left ventricle hypertrophy; MR: Mitral regurgitation; TR: Tricuspid regurgitation.

Table 2 demonstrates changes in echocardiographic findings during transplantation. Transplantation had improved echocardiographic findings. But for few patients they were worsened after transplantation.

**Table 2:** Changes in Echocardiographic Findings During Transplantation

	Not changed	Improved	Worsened
LVEF	8 (26.7%)	17 (56.7%)	5 (16.7%)
LVH	18 (60%)	9 (30%)	3 (10%)
LV dilation	28 (93.3%)	1 (3.3%)	1 (3.3%)
MR	13 (43.3%)	15 (50%)	2 (6.7%)
TR	19 (63.3%)	10 (33.3%)	1 (3.3%)

LVEF: Left ventricle ejection fraction; LVH: Left ventricle hypertrophy; MR: Mitral regurgitation; TR: Tricuspid regurgitation.

## Discussion

Renal transplant resolves many of the cardiac abnormalities associated with chronic renal failure. It is demonstrated that renal transplant could regress echocardiographic findings [9]. Also parameters like LVEF, wall motion and wall thickness are improved during the first six months of post-transplantation [11]. We observed an increased mean LVEF (57.33%) after transplantation in comparison to values before (53.83%), however the increase was not significant. We found that LVEF values were improved in 56.7% of patients, but after transplantation it was reduced in other 16.7% patients. One study, assessing 50 individuals before and 3 months after renal transplantation, de­monstrated significant improvement of the ejection fraction and reduction of the chamber diameters [12]. This increase is reported in other studies as well [10, 13, 14].

It has been shown that LVH initiates along with renal failure, increases with renal failure progression, and it will not be even improved by renal transplantation [15, 16]. Unlike these findings, it is reported that renal transplantation leads to complete resolution of systolic dysfunction, regression of LVH, and improvement of left ventricular dilatation [6]. We observed improvement in LVH in 30% of patients, but it was worsened in 3 cases (10%) after transplantation. Left ventricular dilatation was also improved, but it was seen after transplantation in another patient. Dzemidzic et al [17] in the retrospective study of 30 patients found significant reduction of LVH 1 year after renal transplantation.

We also observed that mitral regurgitation significantly improved after renal transplantation (76.7% to 33.3%); of which all 33.3% had mild mitral regurgitation after transplantation. Tricuspid regurgitation also improved non-significantly. Mitral and tricuspid regurgitation was seen in 6.7% and 3.3% newly after transplantation. Frey and colleagues [18] in evaluating 18 patients with renal transplantation reported valvular abnormalities including mitral and tricuspid in 1 and 3 patients, respectively.

### Conclusion

Our results are indicative of improvement of left ventricle parameters after renal transplantation. Cardiac valve involvement is also regressed. Even in the patients with severe cardiac dysfunction we could observe better performance in a period after renal transplantation.
